# Correction for Canfield et al., “Lytic Bacteriophages Facilitate Antibiotic Sensitization of *Enterococcus faecium”*

**DOI:** 10.1128/aac.00590-25

**Published:** 2025-07-08

**Authors:** Gregory S. Canfield, Anushila Chatterjee, Juliel Espinosa, Mihnea R. Mangalea, Emma K. Sheriff, Micah Keidan, Sara W. McBride, Bruce D. McCollister, Howard C. Hang, Breck A. Duerkop

## AUTHOR CORRECTION

Vol. 65, no. 5, e00143-21, 2021, https://doi.org/10.1128/aac.00143-21. Page 7, Fig. 5A: “L insertion” should read “F insertion.”

Supplemental material: In Fig. S4A, the BLASTP alignment of SagA between *E. faecium* Com12 and Com15 was incorrectly denoted as an L insertion between Y451 and L452. In Table S2, the text in the AA change column for 81R6 was incorrectly labeled as “Leu insertion between Tyr451 & Leu452.” The correct text is given in the revised supplemental file in this correction.

## SUPPLEMENTAL MATERIAL

Supplemental material aac.00590-25-s0001.pdf Fig S1 to S6; Table S1 to S4.

ASM does not own the copyrights to Supplemental Material that may be linked to, or accessed through, an article. The authors have granted ASM a non-exclusive, world-wide license to publish the Supplemental Material files. Please contact the corresponding author directly for reuse.

