# Supplementary material for: Correction for Canfield et al., “Lytic Bacteriophages Facilitate Antibiotic Sensitization of Enterococcus faecium”
Source: Antimicrob Agents Chemother. 2025 Jul 8;69(8):e00590-25. doi: 10.1128/aac.00590-25 (PMC12326954; doi:10.1128/aac.00590-25)
Supplement: Supplemental material — Fig S1 to S6; Table S1 to S4. [file aac.00590-25-s0001.pdf]

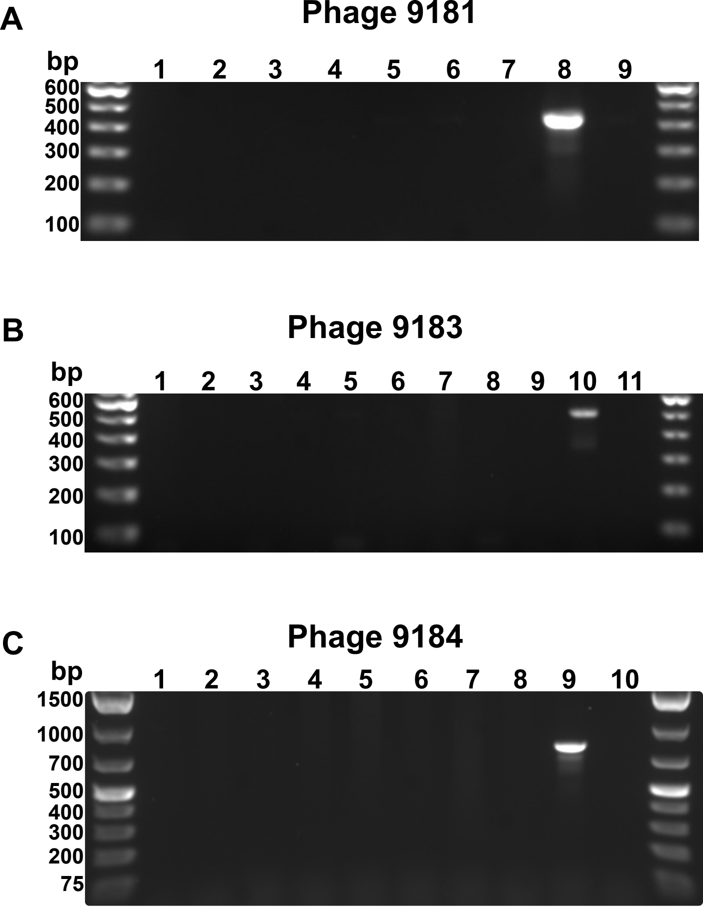
A
Phage 9181
bp
1 2 3 4 5 6 7 8 9
600
500
400
300
200
100
B
Phage 9183
bp
1 2 3 4 5 6 7 8 9 10 11
600
500
400
300
200
100
C
Phage 9184
bp
1 2 3 4 5 6 7 8 9 10
1500
1000
700
500
400
300
200
75

Figure S1

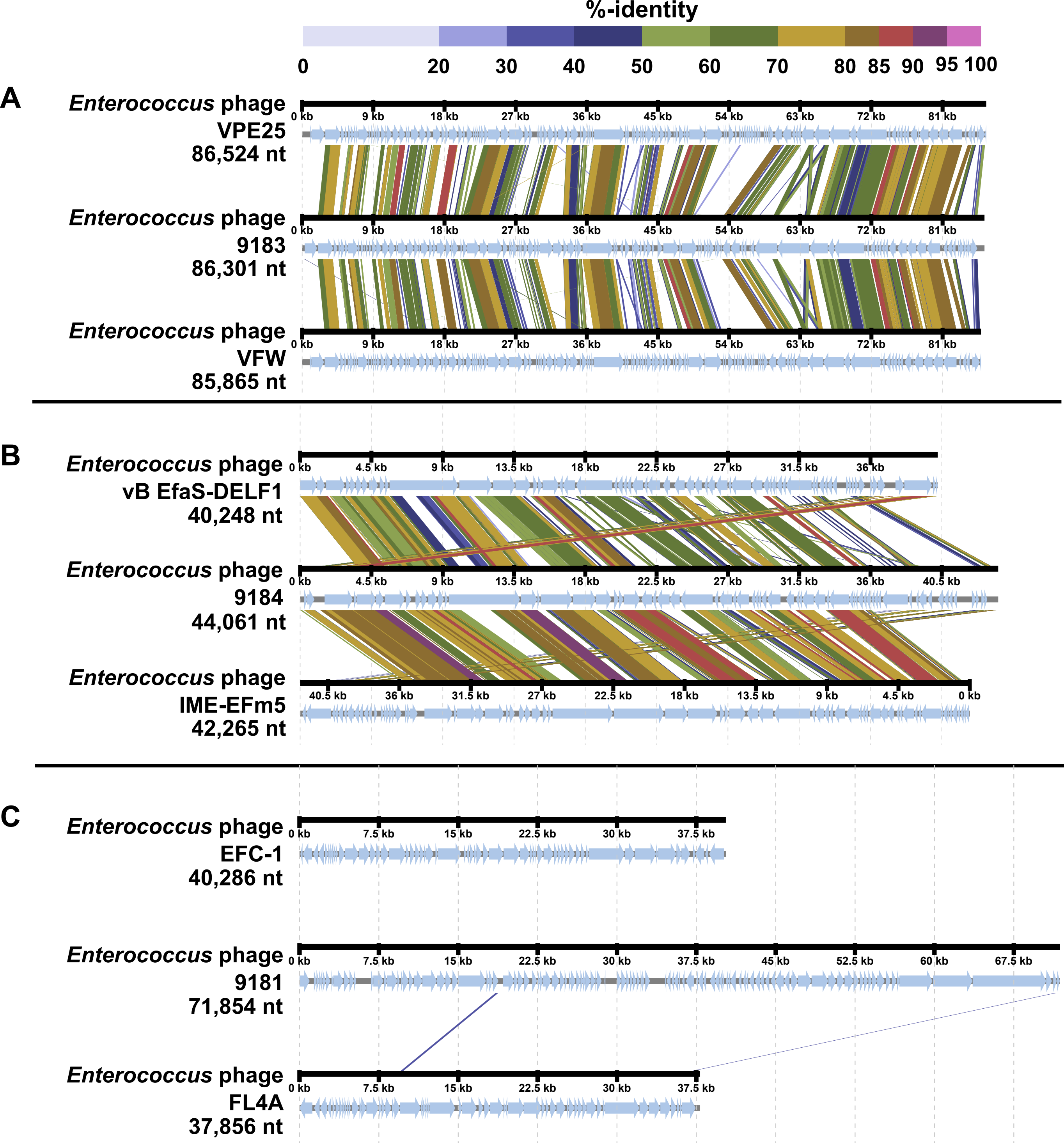

%-identity
0
20
30
40
50
60
70
80
85
90
95
100
A
Enterococcus phage VPE25 86,524 nt
Enterococcus phage 9183 86,301 nt
Enterococcus phage VFW 85,865 nt
B
Enterococcus phage vB EfaS-DELF1 40,248 nt
Enterococcus phage 9184 44,061 nt
Enterococcus phage IME-EFm5 42,265 nt
C
Enterococcus phage EFC-1 40,286 nt
Enterococcus phage 9181 71,854 nt
Enterococcus phage FL4A 37,856 nt

Figure S2

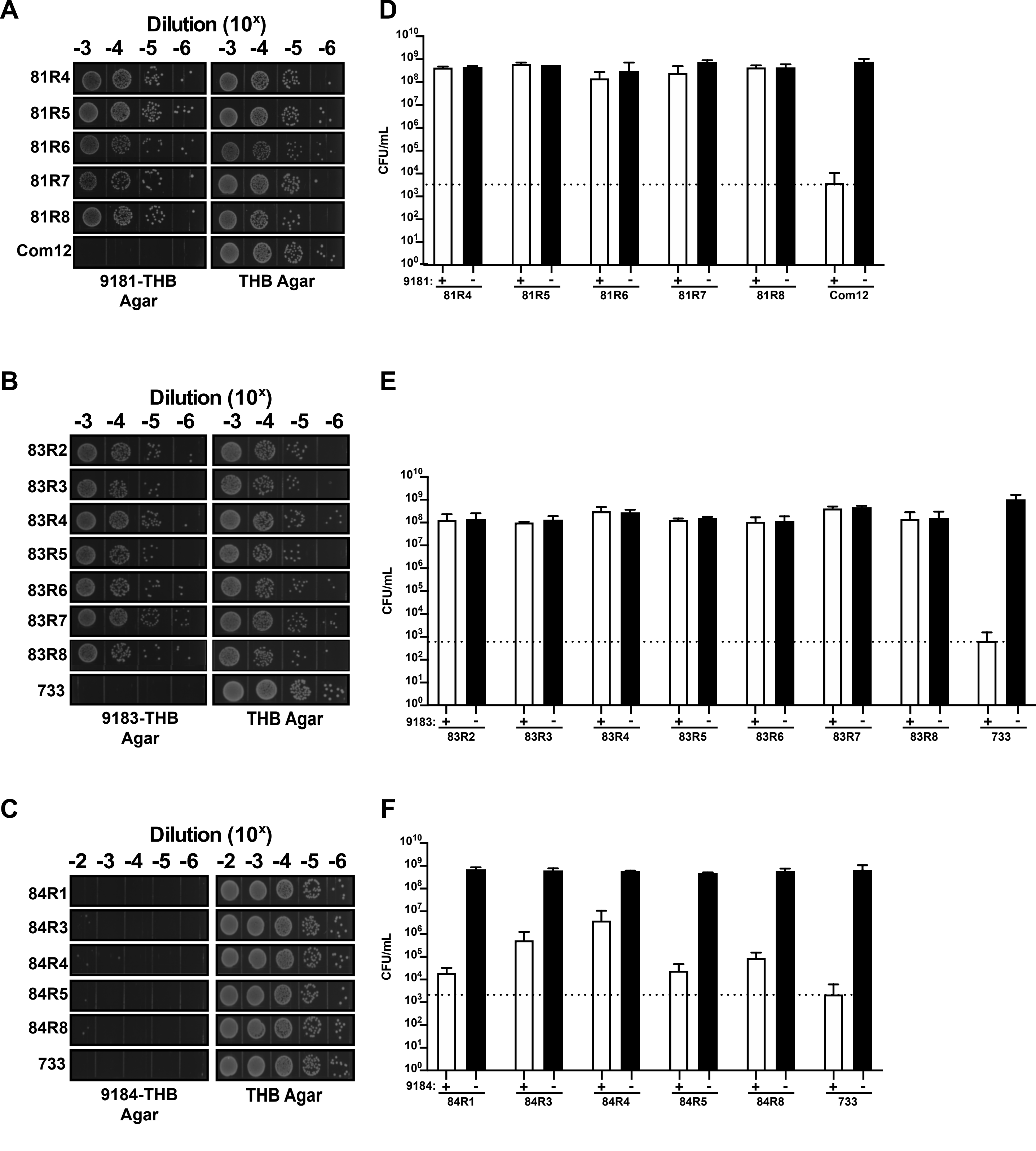

A
Dilution (10x)
-3 -4 -5 -6 -3 -4 -5 -6
81R4
81R5
81R6
81R7
81R8
Com12
9181-THB Agar
THB Agar
D
CFU/mL
9181:
81R4
81R5
81R6
81R7
81R8
Com12
B
Dilution (10x)
-3 -4 -5 -6 -3 -4 -5 -6
83R2
83R3
83R4
83R5
83R6
83R7
83R8
733
9183-THB Agar
THB Agar
E
CFU/mL
9183:
83R2
83R3
83R4
83R5
83R6
83R7
83R8
733
C
Dilution (10x)
-2 -3 -4 -5 -6 -2 -3 -4 -5 -6
84R1
84R3
84R4
84R5
84R8
733
9184-THB Agar
THB Agar
F
CFU/mL
9184:
84R1
84R3
84R4
84R5
84R8
733

Figure S3

A

Identities	Positives	Gaps
513/538(95%)	515/538(95%)	20/538(3%)

```
Com12  1    VKKSLISAVMVCSMTLTAVASPIAAAADDFDSQIQQQDQKIADLKNQQADAQSQIDALES  60
            +KKSLISAVMVCSMTLTAVASPIAAAADDFDSQIQQQDQKIADLKNQQADAQSQIDALES
Com15  1    MKKSLISAVMVCSMTLTAVASPIAAAADDFDSQIQQQDQKIADLKNQQADAQSQIDALES  60

Com12  61   QVSEINTQAQDLLAKQDTLRQESAQLVKDIADLQERIEKREDTIQKQAREAQVSNTSSNY  120
            QVSEINTQAQDLLAKQDTLRQESAQLVKDIADLQERIEKREDTIQKQAREAQVSNTSSNY
Com15  61   QVSEINTQAQDLLAKQDTLRQESAQLVKDIADLQERIEKREDTIQKQAREAQVSNTSSNY  120

Com12  121  IDAVLNADSLADAIGRVQAMTTMVKANNDLMEQQKQDKKAVEDKKAENDAKLKELAENQA  180
            IDAVLNADSLADAIGRVQAMTTMVKANNDLMEQQKQDKKAVEDKKAENDAKLKELAENQA
Com15  121  IDAVLNADSLADAIGRVQAMTTMVKANNDLMEQQKQDKKAVEDKKAENDAKLKELAENQA  180

Com12  181  ALESQKGDLLSKQADLNVLKTSLAAEQATAEDKKADLNRQKAEAEAEQARIREQQRLAEQ  240
            ALESQKGDLLSKQADLNVLKTSLAAEQATAEDKKADLNRQKAEAEAEQARIREQQRLAEQ
Com15  181  ALESQKGDLLSKQADLNVLKTSLAAEQATAEDKKADLNRQKAEAEAEQARIREQQRLAEQ  240

Com12  241  ARQQAAQEKAEKEAREQAEAEAQATQASSTAQSSASEESSAAQSSTTEESSSAAQSSTTE  300
            ARQQAAQEKAEKEAREQAEAEAQATQASS AQSSA+EESSAAQSSTTEESSSAAQSSTTE
Com15  241  ARQQAAQEKAEKEAREQAEAEAQATQASSAAQSSATEESSAAQSSTTEESSSAAQSSTTE  300

Com12  301  ESTTAPESSTTEESTTAPESSTTEESTTVPESSTTEESTTVPESSTTEESTTVPESSTTE  360
            ESTTAPESSTTEESTT PESSTTEESTT PESSTTEESTTVPESSTTEESTTVPES
Com15  301  ESTTAPESSTTEESTTXPESSTTEESTTAPESSTTEESTTVPESSTTEESTTVPES----  356

Com12  361  ESTTVPETSTEESTTPAPTTPSTDQSVDPGNSTGSNATNNT--------TNTTPTPTPSG  412
                    STEESTTPAPTTPSTDQSVDPGNSTGSNATNNT        TNTTPTPTPSG
Com15  357  --------STEESTTPAPTTPSTDQSVDPGNSTGSNATNNTTNTTPXXXTNTTPTPTPSG  408

Com12  413  SVNGAAIVAEAYKYIGTPYVWGGKDPSGFDCSGFTRYVYLQVTGRDIGGWTVPQESAGTK  472
            SVNGAAIVAEAYKYIGTPYVWGGKDPSGFDCSGFTRYVYLQVTGRDIGGWTVPQESAGTK
Com15  409  SVNGAAIVAEAYKYIGTPYVWGGKDPSGFDCSGFTRYVYLQVTGRDIGGWTVPQESAGTK  468

Com12  473  ISVSQAKAGDLLFWGSPGGTYHVAIALGGGQYIHAPQPGESVKVGSVQWFAPDFAVSM  530
            ISVSQAKAGDLLFWGSPGGTYHVAIALGGGQYIHAPQPGESVKVGSVQWFAPDFAVSM
Com15  469  ISVSQAKAGDLLFWGSPGGTYHVAIALGGGQYIHAPQPGESVKVGSVQWFAPDFAVSM  526
```

81R3 and 81R4 (W433G and W433C, respectively)
81R5 (G460D)
81R6 (note: F insertion between Y451 and L452
81R8 (G435V)
Active Site Residues (C443, H494, H506)
Peptidoglycan Clamp Residues (W433 and W462)

B

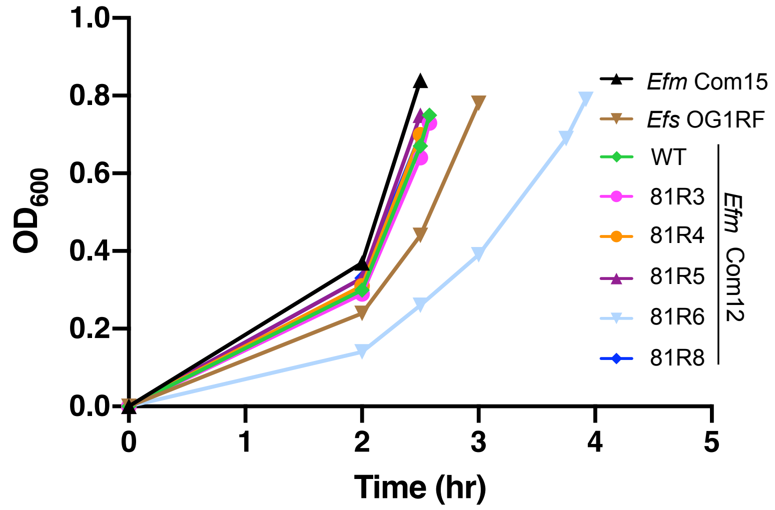

C **D**

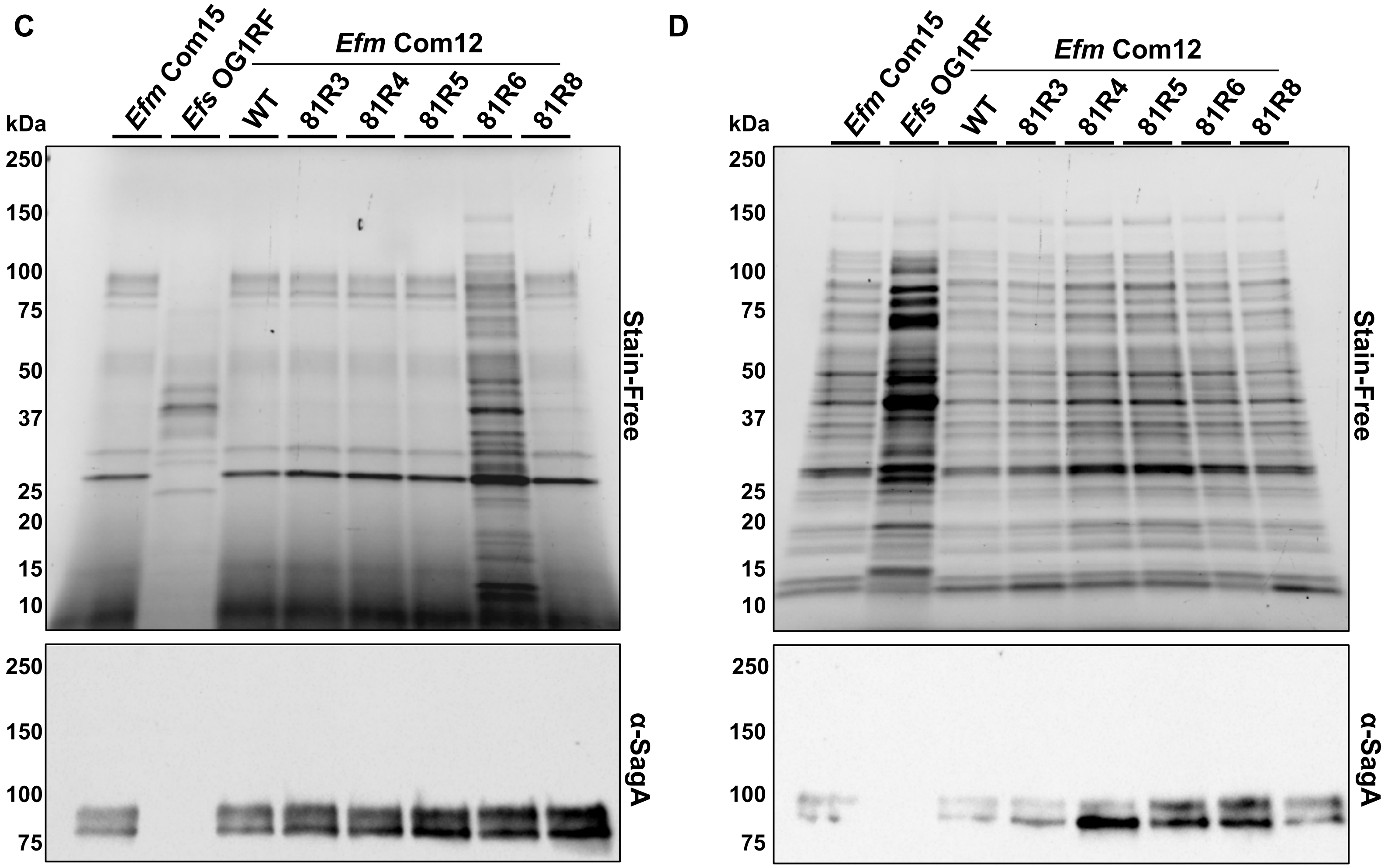

Figure S4

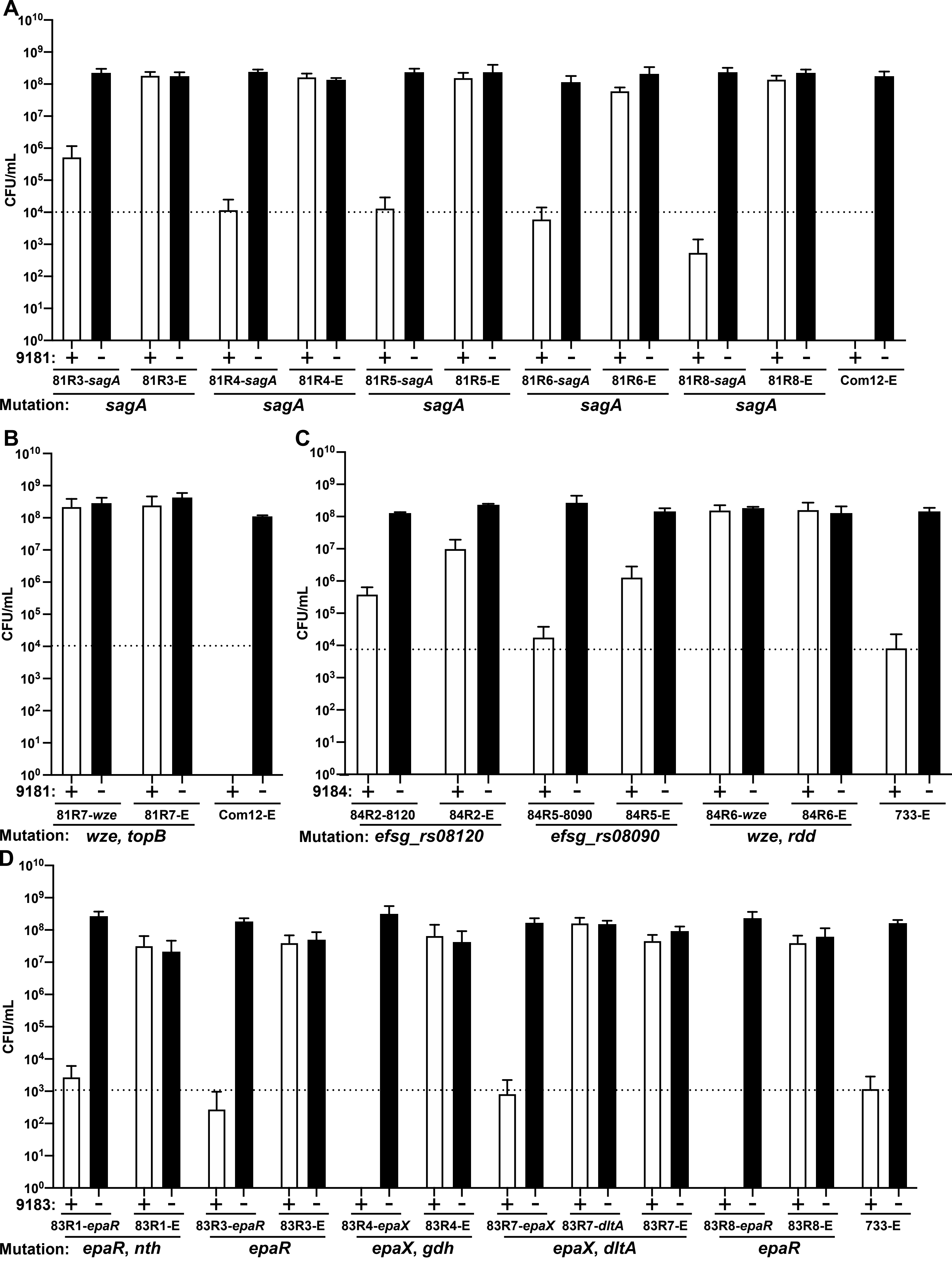

A
B
C
D
CFU/mL
9181:
9184:
9183:
81R3-sagA
81R3-E
81R4-sagA
81R4-E
81R5-sagA
81R5-E
81R6-sagA
81R6-E
81R8-sagA
81R8-E
Com12-E
Mutation:
sagA
81R7-wze
81R7-E
wze, topB
84R2-8120
84R2-E
84R5-8090
84R5-E
84R6-wze
84R6-E
733-E
efsg_rs08120
efsg_rs08090
wze, rdd
83R1-epaR
83R1-E
83R3-epaR
83R3-E
83R4-epaX
83R4-E
83R7-epaX
83R7-dltA
83R7-E
83R8-epaR
83R8-E
epaR, nth
epaR
epaX, gdh
epaX, dltA

Figure S5

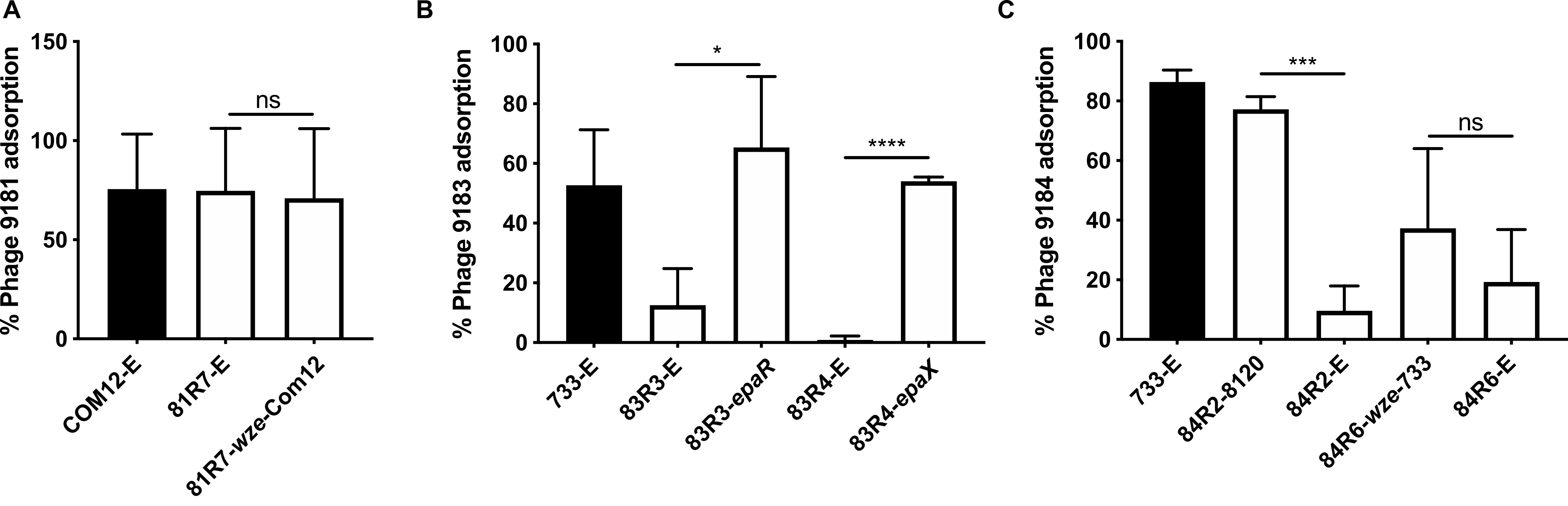
A
% Phage 9181 adsorption
150
100
50
0
ns
COM12-E
81R7-E
81R7-wze-Com12
B
% Phage 9183 adsorption
100
80
60
40
20
0
*
****
733-E
83R3-E
83R3-epaR
83R4-E
83R4-epaX
C
% Phage 9184 adsorption
100
80
60
40
20
0
***
ns
733-E
84R2-8120
84R2-E
84R6-wze-733
84R6-E

Figure S6

Supplemental Figure Legends

Figure S1. PCR screen for phage lysogeny in phage resistant mutants. A molecular weight marker with corresponding band sizes in base pairs (i.e. bp) is shown at the far left and right of each gel image. (A) PCR screen for phage 9181 lysin gene in *E. faecium* or phage 9181 genomic DNA. Lane numbers correspond to the following genomic DNA samples: 1) 81R3, 2) 81R4, 3) 81R5, 4) 81R6, 5) 81R7, 6) 81R8, 7) *E. faecium* Com12, 8) Phage 9181, 9) negative control. (B) PCR screen for phage 9183 integrase gene in *E. faecium* or phage 9183 genomic DNA. Lane numbers correspond to the following genomic DNA samples: 1) 83R1, 2) 83R2, 3) 83R3, 4) 83R4, 5) 83R5, 6) 83R6, 7) 83R7, 8) 83R8, 9) *E. faecium* 1,141,733, 10) Phage 9183, 11) negative control. (C) PCR screen for phage 9184 lysin gene in *E. faecium* or phage 9184 genomic DNA. Lane numbers correspond to the following genomic DNA samples: 1) 84R1, 2) 84R2, 3) 84R3, 4) 84R4, 5) 84R5, 6) 84R6, 7) 84R8, 8) *E. faecium* 1,141,733, 9) phage 9184, 10) negative control.

Figure S2. *Enterococcus faecium* phage orthoclusters. Phage protein coding sequence alignments were performed with nearest neighbors. Colored lines connecting genomes indicate percent protein identity along the length of each genome. (A) Phage 9183 demonstrates protein homology and similar genome organization to its nearest neighbor intra-orthocluster phages (phages VFW and VPE25). (B) Phage 9184 demonstrates proteome homology and similar genome organization to its nearest neighbor intra-orthocluster phages (phages vB EfaS-DELF1 and IME-EFm5). (C) Phage 9181 shows little to no protein homology to its nearest neighbor extra-orthocluster phages (phages EFC-1 and FLA4).

Figure S3. Phage resistant mutants of *E. faecium* following exposure to phages 9181, 9183 and 9184. Phage 9181 (A), 9183 (B), and 9184 (C) susceptibility assays and associated bacterial enumeration of wild type and phage resistant mutants in the presence (white bars) or absence (black bars) of phage (A-F) from three independent experiments. Phage 9181 (A, D) and phage 9183 (B, E) resistant strains exhibit ≥ 5-logs of survival versus *E. faecium* Com12 and 1,141,733 (i.e. 733), respectively. Phage 9184 (C, F) resistant strains exhibit a weak resistance phenotypes. The dotted line indicates the spontaneous mutation threshold

conferring phage resistance observed in the respective wild type host strain of each phage. The threshold was placed to aid in discriminating weak phage resistance phenotypes versus the parental strain.

Figure S4. SagA is conserved in *E. faecium* Com12 and Com15 and SagA is expressed in *sagA* mutants. (A) Displayed is the BLASTP alignment of SagA between Com12 and Com15, showing 95% similarity and strict conservation of peptidoglycan clamp (orange lettering) and active site residues (red lettering). Colored highlights indicate the location of amino acid changes detected in phage 9181 resistant mutants (81R3 and 81R4 – green highlight, 81R5 – yellow highlight, 81R6 – blue highlight, 81R8 – magenta highlight). Specific amino acid changes are noted below the alignment in parentheses next to their respective phage resistant mutant. (B) Growth of *E. faecalis* OG1RF, *E. faecium* Com15, Com12 (WT) and *sagA* mutants (81R3-6; 81R8) are similar in BHI, except for 81R6. (C,D) Displayed is the whole protein fraction (upper panel; Stain-Free) and Western Blot of SagA (lower panel; α-SagA) taken from the exponential phase (OD_{600} ~0.8) supernatants (C) or cell pellets (D) of *E. faecalis* OG1RF, *E. faecium* Com15, Com12 (WT), and *sagA* mutants (81R3-6; 81R8). Protein band sizes are demonstrated to the left of each panel in kilodaltons (kDa).

Figure S5. Complementation restores phage susceptibility in phage resistant mutants. Bacterial enumeration from Phage 9181 (A and B), 9184 (C), and 9183 (D) phage susceptibility assays of wild type and phage resistant mutants complemented with their respective wild type allele or empty vector. Assays were performed in the presence (white bars) or absence (black bars) of phages from two independent experiments. The bars and error bars indicate the average and standard deviation from two independent experiments. The dotted line indicates the spontaneous mutation threshold conferring phage resistance observed in the respective wild type host strain of each phage.

Figure S6. Complementation restores phage adsorption in phage resistant mutants. Percentage phage adsorption of phage resistant mutants, complemented phage resistant mutants, or their parental strains to Phage 9181 (A), 9183 (B), and 9184 (C). Parental and phage resistant mutants were complemented with the empty vector (E; pLZ12a) and compared to their complemented phage resistant mutant strain. Data represent

the mean percent adsorption and standard deviation from three independent experiments. *, $P < 0.05$; ***, $P < 0.001$; ****, $P < 0.0001$; ns, non-significant by unpaired Student's *t* test.

Table S1A. *Enterococcus faecium* phage 9181 genome organization and features

Phage 9181 genome annotation

Feature ID	Gene Start	Gene Stop	Length	Strand	blastp Best-Hit cut-off (0.001) Number	Best-Hit Accession	% Gaps	% Identities	% Positives	E-value	Predicted Function
ORF1	15	959	945	+	N-acetylmuramoyl-L-alanine amidase Enterococcus hirae	OJG47739	6	53	66	7.88E-98	N-acetylmuramoyl-L-alanine amidase
ORF2	1362	1601	240	+							hypothetical protein
ORF3	1605	1853	249	+							hypothetical protein
ORF4	1866	2048	183	+							hypothetical protein
ORF5	2045	2185	141	+							hypothetical protein
ORF6	2199	2483	285	+							hypothetical protein
ORF7	2496	2906	411	+	hypothetical protein EFP29_60 Enterococcus phage EF-P29	APU00246	0	45	68	2.70E-31	hypothetical protein
ORF8	3020	3178	159	+	hypothetical protein Enterococcus casseliflavus	WP_010749468	0	43	61	5.02E-04	hypothetical protein
ORF9	3193	4158	966	+	hypothetical protein X878_0035 Enterococcus phage VD13	YP_009036396	10	47	64	6.70E-70	hypothetical protein
ORF10	4191	4730	540	+							hypothetical protein
ORF11	4727	5146	420	+							hypothetical protein
ORF12	5148	5348	201	+	Enterococcus phage IMEEF1	YP_009603964	3	58	70	8.41E-15	hypothetical protein
ORF13	6777	6971	195	+							hypothetical protein
ORF14	6976	7929	954	+	hypothetical protein Enterococcus phage vB_EfaS_Ef2.2	QBZ69248	1	50	70	7.56E-109	DNA primase
ORF15	7990	8268	279	+							hypothetical protein
ORF16	8268	9251	984	+	hypothetical protein Enterococcus wangshanyuanii	WP_088271390	10	36	53	1.69E-51	Rnl2 family RNA ligase
ORF17	9253	9459	207	+	hypothetical protein Enterococcus faecium	WP_002324739	0	65	76	6.90E-08	hypothetical protein
ORF18	9469	9618	150	+							hypothetical protein
ORF19	9608	9787	180	+							hypothetical protein
ORF20	9787	10323	537	+	hypothetical protein Pseudomonas	WP_092194385	9	39	54	4.74E-20	HNH endonuclease

ORF21	10382	10759	378	+	hypothetical protein Enterococcus gallinarum	WP_142976764 .1	0	79	79	1.00E-20	ribonucleoside-diphosphate reductase
ORF22	10795	11613	819	+	DNA replication protein Enterococcus phage vB_EfaS_EF1c55	QEM41680	2	57	75	3.19E-95	DNA replication initiator protein
ORF23	11610	12983	1374	+	replicative DNA helicase Streptococcus phage SPQS1	YP_008320518	1	59	78	0.00E+00	DNA helicase
ORF24	12994	13791	798	+							hypothetical protein
ORF25	13788	14348	561	+							hypothetical protein
ORF26	14430	14984	555	+	LPS glycosyltransferase Enterococcus phage Entf1	QDB70555	2	33	49	1.26E-05	LPS glycosyltransferase
ORF27	14984	17605	2622	+	DNA polymerase I Enterococcus phage vB_EfaS_Ef2.2	QBZ69269	4	51	66	0.00E+00	DNA polymerase I
ORF28	17671	17919	249	+	hypothetical protein						hypothetical protein
ORF29	17919	18248	330	+							hypothetical protein
ORF30	18271	18801	531	+	hypothetical protein Enterococcus faecalis	WP_016624495	4	26	55	7.23E-12	hypothetical protein
ORF31	18896	19243	348	+	hypothetical protein Enterococcus phage vB_EfaS_Ef2.2	QBZ69253	0	51	67	2.64E-27	HNH endonuclease
ORF32	19227	20279	1053	+	hypothetical protein Enterococcus phage vB_EfaS_EF1c55	QEM41676	1	54	71	4.96E-126	exonuclease
ORF33	20279	20608	330	+							hypothetical protein
ORF34	20602	21168	567	+	crossover junction endodeoxyribonuclease RuvC Enterococcus phage SAP6	YP_009604008	1	44	65	3.79E-44	crossover junction endodeoxyribonuclease RuvC
ORF35	21152	21742	591	+	hypothetical protein Enterococcus phage vB_EfaS_Ef2.2	QBZ69257	5	43	64	8.23E-39	adenylate kinase
ORF36	21717	22448	732	+	hypothetical protein A958_gp43 Enterococcus phage BC611	YP_006488770	3	31	50	4.25E-16	RNA polymerase sigma factor
ORF37	22519	22734	216	+							hypothetical protein
ORF38	22734	22961	228	+							hypothetical protein
ORF39	22980	23780	801	+	hypothetical protein EFP01_126 Enterococcus phage EFP01	APZ82053	0	75	89	1.17E-147	deoxyadenosine kinase deoxyguanosine kinase
ORF40	23837	24568	732	+	PnuC-like NrdR-regulated deoxyribonucleotide	QDB71576	0	73	87	3.66E-126	PnuC-like NrdR-regulated deoxyribonucleotide transporter

					transporter Enterococcus phage vB_OCPT_Ben						
ORF41	24670	25059	390	+							hypothetical protein
ORF42	25056	25277	222	+							Double-strand recombination repair protein
ORF43	25270	25446	177	+							hypothetical protein
ORF44	25458	25661	204	+							hypothetical protein
ORF45	25661	26080	420	+							hypothetical protein
ORF46	26064	26549	486	+							hypothetical protein
ORF47	26543	26797	255	+							hypothetical protein
ORF48	26797	27000	204	+							hypothetical protein
ORF49	26997	27512	516	+							hypothetical protein
ORF50	27509	27958	450	+							hypothetical protein
ORF51	27948	28082	135	+							hypothetical protein
ORF52	28219	28551	333	+	putative HNH endonuclease Lactococcus phage 340	YP_008318136	8	39	51	1.08E-12	HNH endonuclease
ORF53	28806	28910	105	-							hypothetical protein
ORF54	29273	29425	153	-							hypothetical protein
ORF55	29777	29890	114	+							hypothetical protein
ORF56	29993	30400	408	+	hypothetical protein PEf771_147 Enterococcus phage PEf771	QEP29564	10	41	57	4.63E-23	terminal repeat-encoded protein P
ORF57	30416	30883	468	+							hypothetical protein
ORF58	30887	31168	282	+	hypothetical protein EFP01_182 Enterococcus phage EFP01	APZ82109	0	46	73	2.96E-22	hypothetical protein
ORF59	31161	31466	306	+							hypothetical protein
ORF60	31488	31826	339	+	hypothetical protein Enterococcus phage vB_EfaS_PHB08	QBX32893	5	54	65	1.40E-21	hypothetical protein
ORF61	31868	32149	282	+	hypothetical protein EFP29_02 Enterococcus phage EF-P29	APU00188	0	81	89	2.59E-46	hypothetical protein
ORF62	32237	32392	156	+							hypothetical protein
ORF63	32394	32561	168	+							hypothetical protein

ORF64	32673	33122	450	+							hypothetical protein
ORF65	33332	33427	96	-							hypothetical protein
ORF66	33635	33739	105	+							hypothetical protein
ORF67	33761	33880	120	-							hypothetical protein
ORF68	33961	34110	150	-							hypothetical protein
ORF69	34175	34354	180	+							hypothetical protein
ORF70	34395	34508	114	-							hypothetical protein
ORF71	34521	34652	132	-							hypothetical protein
ORF72	34698	35060	363	-	DUF1642 domain-containing protein Enterococcus gilvus	WP_010781701	8	34	59	9.30E-10	DUF1642 domain-containing protein
ORF73	35153	35398	246	-							hypothetical protein
ORF74	35395	35790	396	-							hypothetical protein
ORF75	35803	35952	150	-							hypothetical protein
ORF76	36157	36384	228	-							hypothetical protein
ORF77	36507	37277	771	-							hypothetical protein
ORF78	37264	37518	255	-							hypothetical protein
ORF79	37478	37690	213	-							hypothetical protein
ORF80	37671	38156	486	-							hypothetical protein
ORF81	38149	38421	273	-							hypothetical protein
ORF82	38396	38488	93	-							hypothetical protein
ORF83	38778	39251	474	-							hypothetical protein
ORF84	39253	39684	432	-	DUF1642 domain-containing protein Enterococcus faecium	WP_142972308	11	37	53	4.03E-11	DUF1642 domain-containing protein
ORF85	39684	39974	291	-							hypothetical protein
ORF86	39986	40141	156	-	hypothetical protein A5816_000554 Enterococcus sp. 3G1_DIV0629	OTO28288	0	50	75	2.88E-04	hypothetical protein
ORF87	40141	40302	162	-	hypothetical protein HMPREF9524_01966 Enterococcus faecium TX0133a01	EFR67890	0	43	61	3.88E-04	hypothetical protein

ORF88	40299	40487	189	-	Uncharacterised protein Enterococcus hirae	VTQ86473	0	54	76	9.00E-06	hypothetical protein
ORF89	40499	40705	207	-	hypothetical protein Enterococcus durans	WP_144775119	0	99	99	1.44E-41	hypothetical protein
ORF90	40702	41226	525	-	DUF1642 domain-containing protein Enterococcus faecium	WP_104889537	9	52	65	1.26E-52	DUF1642 domain-containing protein
ORF91	41219	41428	210	-	hypothetical protein Enterococcus faecium	WP_010722442	0	99	100	1.17E-40	hypothetical protein
ORF92	41484	41765	282	-							hypothetical protein
ORF93	41762	41932	171	-							hypothetical protein
ORF94	41922	42161	240	-							hypothetical protein
ORF95	42182	42397	216	-							HNH endonuclease
ORF96	42487	42951	465	-							hypothetical protein
ORF97	43031	43522	492	-							hypothetical protein
ORF98	43519	43902	384	-							hypothetical protein
ORF99	44003	44392	390	-							hypothetical protein
ORF100	44523	44957	435	-							hypothetical protein
ORF101	44960	45754	795	-	dUTP diphosphatase Rummeliibacillus sp. TYF005	WP_124217124	3	45	63	5.46E-32	deoxyuridine 5-triphosphate nucleotidohydrolase
ORF102	45769	46302	534	-	hypothetical protein T548_0137 Lactococcus phage phiL47	YP_009007015	7	28	47	3.93E-05	hypothetical protein
ORF103	46458	47102	645	+	terminase small subunit Enterococcus phage EF-P29	APU00269	3	54	73	9.24E-62	terminase small subunit
ORF104	47099	48376	1278	+	terminase large subunit Enterococcus phage vB_EfaS_Ef7.1	QBZ69408	0	65	80	0.00E+00	terminase large subunit
ORF105	48389	49963	1575	+	phage portal protein Streptococcus phage SPQS1	YP_008320482	3	61	76	0.00E+00	portal protein
ORF106	49976	50722	747	+	hypothetical protein Enterococcus phage vB_EfaS_Ef2.2	QBZ69220	4	33	51	1.74E-28	capsid head morphogenesis protein
ORF107	50824	51468	645	+	DUF4355 domain-containing protein partial *Oceanospirillum linum*	WP_139363748	0	71	87	3.01E-64	DUF4355 domain-containing protein
ORF108	51506	52354	849	+	putative major head protein Enterococcus phage VD13	YP_009036381	1	50	72	2.61E-92	major capsid head protein

ORF109	52357	52875	519	+	major tail protein Staphylococcus phage vB_SauS_IMEP5	ANM47024	2	55	71	2.58E-38	major tail protein
ORF110	52946	53329	384	+	head-tail connector family protein Enterococcus phage EF-P10	AQT27721	2	52	75	6.58E-39	head-tail connector family protein
ORF111	53341	53706	366	+	hypothetical protein Enterococcus phage vB_EfaS_IME198	YP_009218888	4	50	63	4.81E-29	hypothetical protein
ORF112	53694	54071	378	+	hypothetical protein A958_gp18 Enterococcus phage BC611	YP_006488745	2	35	54	7.15E-16	hypothetical protein
ORF113	54082	54567	486	+	hypothetical protein Enterococcus phage vB_EfaS_Ef2.2	QBZ69227	0	64	77	1.43E-58	tail protein
ORF114	54590	55291	702	+	hypothetical protein Enterococcus phage vB_EfaS_Ef2.2	QBZ69228	0	64	79	4.12E-103	Major tail-protein
ORF115	55417	55860	444	+	Ig domain-containing protein Staphylococcus equorum	WP_069832504	7	51	68	1.39E-33	major tail protein
ORF116	55981	56415	435	+	hypothetical protein Enterococcus phage vB_EfaS_IME198	YP_009218892	1	58	76	5.45E-51	hypothetical protein
ORF117	56471	56671	201	+							hypothetical protein
ORF118	56688	59813	3126	+	minor capsid protein Enterococcus phage IMEEF1	YP_009603974	13	42	58	0.00E+00	tail tape measure protein
ORF119	59828	63598	3771	+	tail fiber protein Enterococcus phage Entf1	QDB70491	6	36	57	7.92E-89	tail fiber protein
ORF120	63610	70560	6951	+	BppU family phage baseplate upper protein Enterococcus faecium	WP_016628906	1	87	92	0.00E+00	tail fiber protein
ORF121	70560	71102	543	+							hypothetical protein
ORF122	71222	71554	333	+	holin Enterococcus phage vB_EfaS-DELF1	BBQ04297	0	54	78	5.88E-26	holin
ORF123	71568	71843	276	+	phage holin Enterococcus faecium	WP_086319065	0	67	85	2.79E-39	holin

Table S1B. *Enterococcus faecium* phage 9183 genome organization and features

Phage 9183 genome annotation

Feature ID	Gene Start	Gene Stop	Length	Strand	blastp Best-Hit cut-off (0.001) Number	Best-Hit Accession	% Gaps	% Identities	% Positives	E-value	Predicted Function
ORF1	313	1884	1572	+	AAA family ATPase Pediococcus pentosaceus	WP_055126681	10	30	49	3.60E-51	recombinase recD CDS

ORF2	1982	3919	1938	+	hypothetical protein Enterococcus phage VFW	SCZ83951	0	71	84	0.00E+00	recD-like DNA helicase CDS
ORF3	3912	4460	549	+	None						hypothetical protein
ORF4	4596	4925	330	+	hypothetical protein Enterococcus phage VPE25	SCO93385	0	57	82	3.72E-37	hypothetical protein CDS
ORF5	4922	5386	465	+	hypothetical protein Enterococcus phage VPE25	SCO93386	0	65	83	9.58E-64	hypothetical protein CDS
ORF6	5386	5790	405	+	None						hypothetical protein CDS
ORF7	5787	7073	1287	+	DNA ligase phage-associated Enterococcus phage VPE25	SCO93389	0	70	83	0.00E+00	DNA ligase, phage-associated CDS
ORF8	7070	7258	189	+	None						hypothetical protein CDS
ORF9	7354	7545	192	+	hypothetical protein Enterococcus phage VPE25	SCO93390	0	57	74	6.76E-15	hypothetical protein CDS
ORF10	7532	7747	216	+							hypothetical protein CDS
ORF11	7728	7919	192	+							hypothetical protein CDS
ORF12	8088	8294	207	+							hypothetical protein CDS
ORF13	8446	8703	258	+							hypothetical protein CDS
ORF14	8843	9364	522	+	Lysine decarboxylase family Enterococcus phage VFW	SCZ83963	1	61	80	1.31E-67	Lysine decarboxylase family CDS
ORF15	9379	10026	648	+	Enterococcus phage VPE25	SCO93397	6	59	69	4.10E-79	Purine trans deoxyribosylase Nucleoside deoxyribosyltransferase-I CDS
ORF16	10143	10991	849	+	Deoxyguanosine kinase Enterococcus phage VFW	SCZ83966	0	52	71	1.06E-93	Deoxyguanosine kinase CDS
ORF17	11015	11779	765	+	NrdR-regulated deoxyribonucleotide transporter PnuC-like Enterococcus phage VPE25	SCO93399	0	87	94	5.69E-156	NrdR-regulated deoxyribonucleotide transporter PnuC-like CDS
ORF18	11865	12071	207	+	glutaredoxin-like protein NrdH Enterococcus canis	WP_082703267	3	46	62	5.38E-10	NrdH-like glutaredoxin CDS
ORF19	12146	13189	1044	+	hypothetical protein Enterococcus phage VPE25	SCO93402	1	62	79	7.90E-154	DNA response regulator CDS
ORF20	13278	14126	849	+	hypothetical protein Enterococcus phage VPE25	SCO93403	1	61	78	2.58E-118	HNH endonuclease CDS

ORF21	14119	14712	594	+							hypothetical protein CDS
ORF22	14726	14926	201	+							hypothetical protein CDS
ORF23	15020	15982	963	+	hypothetical protein Enterococcus phage VFW	SCZ83974	2	73	85	5.99E-167	single strand binding protein CDS
ORF24	16056	16523	468	+	hypothetical protein Enterococcus phage VFW	SCZ83975	3	24	54	1.66E-05	hypothetical protein CDS
ORF25	16525	16941	417	+							hypothetical protein CDS
ORF26	17016	18083	1068	+	hypothetical protein Enterococcus phage VPE25	SCO93409	0	85	92	0.00E+00	ATP GTP binding protein CDS
ORF27	18141	18830	690	+							hypothetical protein CDS
ORF28	18820	19020	201	+							hypothetical protein CDS
ORF29	19017	19757	741	+	hypothetical protein Enterococcus phage VPE25	SCO93413	1	51	69	1.65E-79	hypothetical protein CDS
ORF30	19837	20013	177	+	hypothetical protein SSG_02354 Enterococcus faecium EnGen0190	EOH43274	0	82	91	7.54E-28	hypothetical protein CDS
ORF31	20023	20259	237	+	hypothetical protein Peptostreptococcus sp. MV1	WP_052045454	10	55	71	7.14E-21	hypothetical protein CDS
ORF32	20351	20854	504	+	phage protein Enterococcus phage VPE25	SCO93419	0	70	83	7.69E-82	hypothetical protein CDS
ORF33	20851	22431	1581	+	Replicative DNA helicase DnaB Enterococcus phage VPE25	SCO93420	0	82	93	0.00E+00	Replicative DNA helicase DnaB CDS
ORF34	22444	23493	1050	+	Phage toprim domain containing protein YorJ B.subtilis homolog Enterococcus phage VPE25	SCO93421	0	70	86	0.00E+00	DNA primase CDS
ORF35	23505	25328	1824	+	Single-stranded-DNA-specific exonuclease RecJ Enterococcus phage VPE25	SCO93422	2	60	76	0.00E+00	Single-stranded-DNA-specific exonuclease RecJ CDS
ORF36	25413	25988	576	+	hypothetical protein Enterococcus phage VPE25	SCO93423	6	36	59	1.01E-28	hypothetical protein CDS
ORF37	26176	26355	180	+							hypothetical protein CDS
ORF38	26352	26690	339	+							hypothetical protein CDS
ORF39	26695	27207	513	+							hypothetical protein CDS
ORF40	27245	27394	150	+							hypothetical protein CDS

ORF41	27472	27702	231	+							hypothetical protein CDS
ORF42	27727	27870	144	+	hypothetical protein Enterococcus phage VPE25	SCO93428	0	67	87	4.92E-14	hypothetical protein CDS
ORF43	28004	28522	519	+	hypothetical protein Enterococcus phage VPE25	SCO93430	7	47	64	3.36E-35	DTMP kinase, thymidylate kinase CDS
ORF44	28523	29356	834	+	Thymidylate synthase Enterococcus phage VPE25	SCO93431	1	55	73	5.74E-103	Thymidylate synthase CDS
ORF45	29340	29756	417	+	Deoxyuridine 5-triphosphate nucleotidohydrolase Enterococcus phage VPE25	SCO93432	0	78	90	1.59E-75	Deoxyuridine 5-triphosphate nucleotidohydrolase CDS
ORF46	29756	30100	345	+	HAD hydrolase family protein Longirhabdus pacifica	WP_128894611	3	50	68	5.41E-31	HAD hydrolase family protein CDS
ORF47	30097	31731	1635	+	hypothetical protein A2138_23700 Deltaproteobacteria bacterium RBG_16_71_12	OGQ17127	7	33	52	4.86E-83	nucleoside-diphosphate-sugar pyrophosphorylase-like protein CDS
ORF48	31736	32956	1221	+	hypothetical protein CBD54_002560 Alphaproteobacteria bacterium TMED194	RPH07865	6	30	50	5.41E-45	hypothetical protein CDS
ORF49	33043	33567	525	+	Ribonuclease HI Enterococcus phage VPE25	SCO93434	0	87	93	1.14E-107	Ribonuclease HI CDS
ORF50	33564	34535	972	+	Nucleotide sugar synthetase-like protein Enterococcus phage VPE25;	SCO93435	1	48	68	5.98E-98	Nucleotide sugar synthetase-like protein CDS
ORF51	34525	34779	255	+							hypothetical protein CDS
ORF52	34772	35380	609	+	hypothetical protein Enterococcus phage VPE25	SCO93436	1	52	68	1.03E-19	Thymidine kinase CDS
ORF53	35460	39314	3855	+	DNA polymerase III alpha subunit Enterococcus phage VFW	SCZ84010	0	78	89	0.00E+00	DNA polymerase III alpha subunit CDS
ORF54	39381	39644	264	+	hypothetical protein Enterococcus phage VPE25	SCO93444	1	35	57	9.42E-08	hypothetical protein CDS
ORF55	39641	39919	279	+							hypothetical protein CDS
ORF56	40197	40610	414	-							hypothetical protein CDS

ORF57	40850	41422	573	+	hypothetical protein Enterococcus phage VFW	SCZ84015	9	32	50	2.72E-07	hypothetical protein CDS
ORF58	41412	41699	288	+							hypothetical protein CDS
ORF59	41744	41962	219	+	hypothetical protein Bacillus cereus	WP_073526565	3	70	80	3.62E-27	DUF2829 domain-containing protein CDS
ORF60	41962	42705	744	+	Deoxyuridine 5-triphosphate nucleotidohydrolase Enterococcus phage VPE25	SCO93451	3	58	72	1.68E-83	Deoxyuridine 5-triphosphate nucleotidohydrolase CDS
ORF61	42706	43005	300	+							hypothetical protein CDS
ORF62	42995	43594	600	+	Guanylate kinase Enterococcus phage VPE25	SCO93453	5	41	65	5.56E-40	Guanylate kinase CDS
ORF63	43595	44179	585	+	non-essential protein Enterococcus phage VPE25	SCO93454	0	75	90	5.21E-103	RusA family crossover junction endodeoxyribonuclease CDS
ORF64	44148	44531	384	+							hypothetical protein CDS
ORF65	44946	45575	630	+	hypothetical protein Enterococcus phage VPE25	SCO93459	1	53	74	3.72E-73	sigma-70 family RNA polymerase sigma factor CDS
ORF66	45677	47614	1938	+	DNA gyrase subunit B Enterococcus phage VPE25	SCO93463				0.00E+00	Topoisomerase IV subunit B CDS
ORF67	47709	47966	258	+							hypothetical protein CDS
ORF68	47959	49962	2004	+	DNA gyrase subunit A Enterococcus phage VFW	SCZ84033	2	60	77	0.00E+00	DNA topoisomerase IV subunit A CDS
ORF69	50114	50290	177	+							hypothetical protein CDS
ORF70	50393	50623	231	+							hypothetical protein CDS
ORF71	50820	51230	411	+							hypothetical protein CDS
ORF72	51220	51531	312	+							hypothetical protein CDS
ORF73	51528	52100	573	+							hypothetical protein CDS
ORF74	52217	52405	189	+							hypothetical protein CDS
ORF75	52431	52904	474	+							hypothetical protein CDS
ORF76	52921	53103	183	+							hypothetical protein CDS
ORF77	53139	54116	978	-	prophage LambdaBa02 site-specific recombinase phage integrase family Enterococcus phage VFW	SCZ84050	0	86	92	0.00E+00	site-specific recombinase phage integrase family CDS

ORF78	54176	55339	1164	-	N-acetylmuramoyl-L-alanine amidase Enterococcus phage VPE25	SCO93486	11	48	59	1.91E-102	N-acetylmuramoyl-L-alanine amidase CDS
ORF79	55451	55810	360	-	hypothetical protein Enterococcus phage VPE25	SCO93487	0	67	84	8.04E-52	holin CDS
ORF80	55810	56181	372	-	hypothetical protein Enterococcus phage VPE25	SCO93488	0	72	84	8.92E-40	hypothetical protein CDS
ORF81	56159	56557	399	-	hypothetical protein Enterococcus phage VPE25	SCO93489	0	61	83	8.36E-57	hypothetical protein CDS
ORF82	56564	56698	135	-							hypothetical protein CDS
ORF83	56701	57186	486	-	hypothetical protein Enterococcus faecalis	WP_010774487	12	42	56	1.77E-18	hypothetical protein CDS
ORF84	57207	60089	2883	-	BppU family phage baseplate upper protein Enterococcus faecium	WP_104807894	4	53	68	0.00E+00	BppU family phage baseplate upper protein CDS
ORF85	60103	64134	4032	-	hypothetical protein Enterococcus faecium	WP_142972363	6	48	59	4.22E-150	minor tail protein CDS
ORF86	64164	66476	2313	-	hypothetical protein Enterococcus faecalis	WP_057086899	8	48	63	0.00E+00	Phage endopeptidase, tail-spike protein CDS
ORF87	66473	67264	792	-	hypothetical protein Enterococcus phage VPE25	SCO93493	0	62	78	9.10E-121	Phage tail protein CDS
ORF88	67277	71119	3843	-	Phage tail length tape-measure protein Enterococcus phage VPE25	SCO93494	2	54	72	0.00E+00	Phage tail length tape-measure protein CDS
ORF89	71373	71705	333	-	hypothetical protein Enterococcus phage VPE25	SCO93496	0	76	90	1.98E-53	hypothetical protein CDS
ORF90	71872	72480	609	-	Phage major tail protein phi13 Enterococcus phage VFW	SCZ84062	0	89	92	2.55E-107	Phage major tail protein, phage 13 family CDS
ORF91	72504	72881	378	-	hypothetical protein Enterococcus phage VPE25	SCO93498	0	84	90	2.68E-69	hypothetical protein CDS
ORF92	72884	73333	450	-	hypothetical protein Enterococcus phage VPE25	SCO93499	0	71	80	4.30E-70	Phage head-tail joining protein, HK97 gp10 family CDS
ORF93	73326	73679	354	-	hypothetical protein Enterococcus phage VPE25	SCO93500	0	78	92	1.49E-60	Phage head-tail adaptor protein CDS
ORF94	73683	74060	378	-	hypothetical protein Enterococcus phage VPE25	SCO93501	0	72	85	9.18E-61	Phage head-tail connector protein CDS

ORF95	74200	75069	870	-	prophage pi2 protein 34 Enterococcus phage VPE25	SCO93502	0	68	81	6.18E-141	Prophage pi2 protein 34 CDS
ORF96	75270	76475	1206	-	hypothetical protein Enterococcus phage VFW	SCZ84068	1	79	87	0.00E+00	Phage major capsid protein CDS
ORF97	76465	77673	1209	-	hypothetical protein Enterococcus phage VFW	SCZ84069	3	61	71	3.51E-150	Phage prohead protease, HK97 family CDS
ORF98	77688	78965	1278	-	hypothetical protein Enterococcus phage VPE25	SCO93505	0	77	89	0.00E+00	Phage portal protein CDS
ORF99	78978	80684	1707	-	hypothetical protein Enterococcus phage VFW	SCZ84071	0	85	92	0.00E+00	Phage terminase large subunit CDS
ORF100	81115	81606	492	-	Phage-related protein Enterococcus phage VPE25	SCO93509	0	86	96	4.62E-101	Phage terminase small subunit, P27 family CDS
ORF101	81609	82220	612	-	hypothetical protein Bacillus	WP_063263132	14	38	51	5.16E-12	GIY-YIG homing endonuclease CDS
ORF102	82217	82636	420	-	hypothetical protein Enterococcus phage VPE25	SCO93510	3	59	78	1.57E-54	HNH endonuclease CDS
ORF103	82611	82769	159	-							hypothetical protein CDS
ORF104	82955	83212	258	+							hypothetical protein CDS
ORF105	83214	83657	444	+							hypothetical protein CDS
ORF106	83654	84031	378	+							hypothetical protein CDS
ORF107	84028	84219	192	+							hypothetical protein CDS
ORF108	84216	84497	282	+							hypothetical protein CDS
ORF109	84526	85398	873	+	hypothetical protein Enterococcus phage VPE25	SCO93512	3	42	62	2.53E-63	hypothetical protein CDS

Table S1C. *Enterococcus faecium* phage 9184 genome organization and features

Phage 9184 genome annotation

Feature ID	Gene Start	Gene Stop	Length	Strand	blastp Best-Hit cut-off (0.001) Number	Best-Hit Accession	% Gaps	% Identities	% Positives	E-value	Predicted Function
ORF1	260	439	180	+	hypothetical protein Enterococcus phage vB_EfaS-DELF1	BBQ04339	0	78	85	2.55E-25	hypothetical protein
ORF2	444	902	459	+	terminase small subunit Enterococcus phage IME-EFm5	YP_009200920	1	78	86	3.11E-76	terminase small subunit

ORF3	1545	3323	1779	+	terminase large subunit Enterococcus phage IME-EFm1	YP_009042651	0	94	98	0	terminase large subunit
ORF4	3391	3567	177	+	sensor histidine kinase Enterococcus phage IME-EFm1	YP_009042652	0	97	98	2.96E-30	sensor histidine kinase
ORF5	3571	4776	1206	+	portal protein Enterococcus phage IME-EFm5	YP_009200917	0	81	90	0.00E+00	portal protein
ORF6	4784	5278	495	+	prohead protease Enterococcus phage IME-EFm5	YP_009200916	0	95	99	1.88E-105	prohead protease
ORF7	5348	6595	1248	+	capsid protein Enterococcus phage Nonaheksakonda	AZS06457	6	61	76	3.10E-162	capsid protein
ORF8	6675	7013	339	+	head-tail joining protein Enterococcus phage IME-EFm5	YP_009200913	0	79	91	2.64E-61	head-tail connector protein
ORF9	6943	7278	336	+	head-tail adaptor protein Enterococcus phage IME-EFm1	YP_009042657	0	95	96	7.59E-71	head-tail adaptor protein
ORF10	7280	7651	372	+	head-tail joining protein Enterococcus phage IME-EFm5	YP_009200912	0	89	93	4.06E-74	head-tail joining protein
ORF11	7651	8016	366	+	head-tail joining protein Enterococcus phage IME-EFm5	YP_009200911	0	87	94	1.97E-71	head-tail joining protein
ORF12	8089	8649	561	+	major tail protein Enterococcus phage IME-EFm1	YP_009042660	1	87	95	1.35E-113	major tail protein
ORF13	8708	9088	381	+	putative tail tape measure chaperone protein Enterococcus phage IME-EFm5	YP_009200909	0	81	93	2.90E-56	tail tape measure protein
ORF14	9121	9318	198	+	tail tape measure chaperone protein Enterococcus phage IME-EFm1	YP_009042662	0	89	97	2.11E-35	tail tape measure chaperone protein
ORF15	9383	13873	4491	+	transglycosylase SLT domain-containing protein Enterococcus durans	WP_119219106	1	75	85	0.00E+00	tail length tape-measure protein
ORF16	13945	14967	1023	+	minor tail protein Enterococcus phage IME-EFm5	YP_009200906	0	92	97	0.00E+00	minor tail protein
ORF17	14954	17174	240	+	minor tail protein Enterococcus phage IME-EFm5	YP_009200906	0	71	81	0.00E+00	BppU family phage baseplate upper protein
ORF18	17247	18650	1404	+	minor tail protein Enterococcus phage IME-EFm5	YP_009200905	1	75	86	0.00E+00	minor tail protein

ORF19	18665	19651	987	+	tail assembly protein Enterococcus phage IME-EFm5	YP_009200904	2	58	72	1.74E-110	tail assembly protein
ORF20	19830	20108	279	+	holin Enterococcus phage IME-EFm1	YP_009042670	0	97	99	1.24E-57	holin
ORF21	20122	20403	282	+	holin Enterococcus phage IME-EFm5	YP_009200902	0	98	99	2.26E-58	holin
ORF22	20420	21445	1026	+	N-acetylmuramoyl-L-alanine amidase Enterococcus phage IME-EFm5	YP_009200901	0	95	96	0.00E+00	N-acetylmuramoyl-L-alanine amidase
ORF23	21524	22219	696	-	hypothetical protein EFm5_30 Enterococcus phage IME-EFm5	YP_009200900	0	90	95	3.40E-150	Deoxyguanosine kinase
ORF24	22517	23347	831	-	hypothetical protein phiSHEF2_24 Enterococcus phage phiSHEF2	YP_009613304	0	63	80	8.82E-24	DNA polymerase
ORF25	23314	23958	645	-	hypothetical protein Streptococcus pyogenes	WP_136291116	6	33	57	5.20E-27	ABC transporter ATP-binding protein
ORF26	24003	26033	2031	-	DNA polymerase Enterococcus phage vB_EfaS-DELF1	BBQ04302	1	62	77	0.00E+00	DNA polymerase
ORF27	26142	26348	207	-	hypothetical protein Enterococcus phage vB_EfaS-DELF1	BBQ04303	0	65	78	1.57E-24	hypothetical protein
ORF28	26455	27240	786	-	hypothetical protein IME_032 Enterococcus phage IME-EFm1	YP_009042680	5	50	71	3.54E-71	hypothetical protein
ORF29	27299	27517	219	-							hypothetical protein
ORF30	27514	28323	810	-	hypothetical protein EFm5_22 Enterococcus phage IME-EFm5	YP_009200892	0	78	87	1.61E-146	Protein of unknown function DUF1351
ORF31	28313	28483	171	-	hypothetical protein IME_035 Enterococcus phage IME-EFm1	YP_009042683	0	89	93	9.29E-26	hypothetical protein
ORF32	28480	28701	222	-							hypothetical protein
ORF33	28701	28928	228	-	hypothetical protein EFm5_20 Enterococcus phage IME-EFm5	YP_009200890	0	92	97	1.47E-42	hypothetical protein
ORF34	29107	29436	330	-	hypothetical protein IME_030 Enterococcus phage IME-EFm1	YP_009042678	3	51	72	9.10E-32	hypothetical protein
ORF35	29474	30235	762	-	metallo-beta-lactamase domain protein Enterococcus phage vB_EfaS-DELF1	BBQ04313	0	74	86	1.26E-131	Metallo-beta-lactamase domain protein

ORF36	30210	30680	471	-	HNH homing endonuclease-like protein Enterococcus phage IME-EFm5	YP_009200887	0	79	92	1.55E-90	HNH homing endonuclease-like protein
ORF37	30670	31161	492	-	hypothetical protein IME_041 Enterococcus phage IME-EFm1	YP_009042689	2	61	73	1.33E-55	hypothetical protein
ORF38	31301	31501	201	-	hypothetical protein EFm5_15 Enterococcus phage IME-EFm5	YP_009200885	0	86	97	4.58E-36	hypothetical protein
ORF39	31501	31680	180	-	hypothetical protein EFm5_14 Enterococcus phage IME-EFm5	YP_009200884	0	66	76	6.47E-18	hypothetical protein
ORF40	31677	32417	741	-	DNA replication protein Enterococcus phage IME-EFm5	YP_009200883	0	82	91	2.59E-150	bifunctional DNA primase-polymerase
ORF41	32414	33154	741	-	adenine-specific methyltransferase Enterococcus phage vB_EfaS_Ef5.4	QBZ69846	2	63	79	7.63E-108	DNA methylase
ORF42	33210	33368	159	-	hypothetical protein Enterococcus phage vB_EfaS-DELF1	BBQ04319	7	71	82	1.93E-17	hypothetical protein
ORF43	33365	34642	1278	-	helicase Enterococcus phage IME-EFm5	YP_009200880	0	90	95	0.00E+00	DNA helicase
ORF44	34639	34914	276	-	hypothetical protein EFm5_08 Enterococcus phage IME-EFm5	YP_009200878	2	77	90	1.21E-44	putative RNA-binding protein
ORF45	34914	35030	117	-	hypothetical protein EFm5_07 Enterococcus phage IME-EFm5	YP_009200877	0	61	79	4.99E-07	hypothetical protein
ORF46	35027	35194	168	-	hypothetical protein EFm5_06 Enterococcus phage IME-EFm5	YP_009200876	0	73	85	3.36E-20	hypothetical protein
ORF47	35187	35537	351	-	endonuclease Enterococcus phage IME-EFm5	YP_009200875	0	93	98	5.11E-74	Endonuclease
ORF48	35534	35749	216	-							hypothetical protein
ORF49	35746	35973	228	-	hypothetical protein EFm5_03 Enterococcus phage IME-EFm5	YP_009200873	1	66	83	9.04E-21	hypothetical protein
ORF50	35963	36055	93	-							hypothetical protein
ORF51	36055	36252	198	-							hypothetical protein
ORF52	36249	36464	216	-							hypothetical protein
ORF53	36461	36658	198	-							hypothetical protein

ORF54	36735	38357	1623	-	DNA primase Enterococcus phage vB_EfaS-DELF1	BBQ04327	9	31	51	3.86E-50	DNA primase
ORF55	38404	38607	204	-	hypothetical protein Enterococcus phage vB_EfaS-DELF1	BBQ04330	6	60	72	1.27E-12	hypothetical protein
ORF56	38678	38860	183	-	hypothetical protein EFm5_68 Enterococcus phage IME-EFm5	YP_009200938	0	82	95	1.09E-28	putative swarming motility protein
ORF57	38853	39032	180	-	hypothetical protein EFm5_67 Enterococcus phage IME-EFm5	YP_009200937	0	64	83	3.69E-19	hypothetical protein
ORF58	39032	39535	504	-	hypothetical protein EFm5_66 Enterococcus phage IME-EFm5	YP_009200936	0	75	86	4.21E-28	tail length tape-measure protein
ORF59	39601	39831	231	-	hypothetical protein CUN38_04900 Enterococcus faecium	PQC93482	0	80	89	2.27E-34	hypothetical protein
ORF60	39831	40040	210	-							hypothetical protein
ORF61	40056	40238	183	-	hypothetical protein Enterococcus faecalis	WP_033659461	0	58	77	5.57E-14	hypothetical protein
ORF62	40251	40613	363	-	hypothetical protein IME_057 Enterococcus phage IME-EFm1	YP_009042705	0	71	87	4.63E-42	hypothetical protein
ORF63	40625	40951	327	-	hypothetical protein IME_059 Enterococcus phage IME-EFm1	YP_009042707	0	82	91	1.40E-58	DUF1140 protein
ORF64	40945	41178	234	-	hypothetical protein IME_060 Enterococcus phage IME-EFm1	YP_009042708	1	60	81	5.08E-24	hypothetical protein
ORF65	41234	41467	234	-							hypothetical protein
ORF66	41524	41826	303	-							hypothetical protein
ORF67	42246	42410	165	+							hypothetical protein
ORF68	42440	42664	225	+							hypothetical protein
ORF69	42661	42843	183	+							hypothetical protein
ORF70	42809	42988	180	+							hypothetical protein
ORF71	42999	43190	192	+	hypothetical protein EFm5_55 Enterococcus phage IME-EFm5	YP_009200925	0	71	89	2.29E-26	hypothetical protein
ORF72	43190	43366	177	+	hypothetical protein IME_069 Enterococcus phage IME-EFm1	YP_009042717	10	78	86	3.14E-23	hypothetical protein
ORF73	43565	43942	378	+	HNH endonuclease Enterococcus phage IME-EFm5	YP_009200923	0	89	96	4.02E-76	HNH endonuclease

Table S2A. Spontaneous, non-synonymous mutations in secreted antigen A (SagA) gene promote phage 9181 resistance

Phage 9181 Resistant Mutant	Com12 Contig Number	Contig Position	Variation Type	Variation Frequency (%)	Fold Coverage	Gene locus: SNP/Indel	AA change	Putative function
81R3	NZ_GG670310	172959	SNP	100	160	EFVG_RS16270:313G ⇒ T	Trp433 ⇒ Leu	SagA
81R4	NZ_GG670310	172960	SNP	100	279	EFVG_RS16270: 314G ⇒T	Trp433 ⇒ Cys	SagA
81R5	NZ_GG670310	173040	SNP	100	119	EFVG_RS16270:394G ⇒A	Gly460 ⇒ Asp	SagA
81R6	NZ_GG670310	173014	Insertion	71.79	156	EFVG_RS16270:368-370 CTT insertion	Phe insertion between Tyr451 & Leu452	SagA
81R7	NZ_GG670306	96441	SNP	100	114	EFVG_RS05165:325G ⇒ T	Gly109 ⇒ Trp	DNA topoisomerase III
81R7	NZ_GG670306	418448	SNP	100	115	EFVG_RS06695: 499C ⇒ T	Pro167 ⇒ Ser	Capsule tyrosine-protein kinase (*wze*)
81R8	NZ_GG670310	172965	SNP	99.43	176	EFVG_RS16270: 319G ⇒ T	Gly435 ⇒ Val	SagA

SNP – Single Nucleotide Polymorphism; Indel – Insertion/Deletion; SagA – secreted antigen A; AA – amino acid

Table S2B. Spontaneous, non-synonymous mutations in the *epa* locus promotes phage 9183 resistance

Phage 9183 Resistant Mutant	1,141,733 Contig Number	Contig Position	Variation Type	Variation Frequency (%)	Fold Coverage	Gene locus: SNP/Indel	AA change	Putative function
83R1	NZ_GG688464	157195	SNP #1	100	79	EFSG_RS16205: 1012C ⇒ T	Arg338 ⇒ Cys	Polyprenyl Glycosylphosphotransferase (*epaR*)
83R1	NZ_GG688461	1077065	SNP #2	100	6	EFSG_RS11135: 28G ⇒ T	Ala10 ⇒ Ser	Endonuclease III (*nth*)
83R2	No mutation detected							
83R3	NZ_GG688464	156490	Deletion	100	189	EFSG_RS16205: Deletion of 309T	Phe103 Frameshift	Polyprenyl Glycosylphosphotransferase (*epaR*)
83R4	NZ_GG688462	47014	SNP	100	167	EFSG_RS12860: 741C ⇒T	Trp104 ⇒ Cys	Gluconate 5-Dehydrogenase (*gdh*)
83R4	NZ_GG688464	161642	Deletion	88.29	111	EFSG_RS16230: Deletion of 197A	Asn66 Frameshift	TarS-like Glycosyltransferase (*epaX*)
83R5	NZ_GG688461	891434	SNP #1	100	78	EFSG_RS10305: 5A ⇒ G	Glu2 ⇒ Gly	General Stress Response Protein A (*gnsA*)
83R5	NZ_GG688464	156807	Deletion	95.83	48	EFSG_RS16205: Deletion of 630A	Glu211 Frameshift	Polyprenyl Glycosylphosphotransferase (*epaR*)
83R5	NZ_GG688461	332892	SNP #2	43.75	80	EFSG_RS07565: 985C ⇒ T	Leu329 ⇒ Phe	SorC family transcriptional regulator (*sorC*)
83R6	NZ_GG688464	157113	SNP	100	75	EFSG_RS16205: 930G ⇒ T	Met310 ⇒ Ile	Polyprenyl Glycosylphosphotransferase (*epaR*)
83R7	NZ_GG688464	67506	SNP	100	74	EFSG_RS15760: 22G ⇒ T	Glu8 ⇒ Stop	D-alanine--poly(phosphoribitol) ligase subunit (*dltA*)

83R7	NZ_GG688464	161642	Deletion	92.05	88	EFSG_RS16230: Deletion of 197A	Asn66 Frameshift	Tar-S-like Glycosyltransferase (*epaX*)
83R8	NZ_GG688464	157127	SNP	100	59	EFSG_RS16205: 944A ⇒ G	Glu315 ⇒ Gly	Polyprenyl Glycosylphosphotransferase (*epaR*)
SNP – Single Nucleotide Polymorphism; Indel – Insertion/Deletion; AA – Amino Acid								

Table S2C. Spontaneous, non-synonymous mutations in the capsule locus and *rdd* (*efsg_rs09545*) genes promotes phage 9184 resistance

Phage 9184 Resistant Mutant	1,141,733 Contig Number	Contig Position	Variation Type	Variation Frequency (%)	Fold Coverage	Gene locus: SNP/Indel	AA change	Putative function
84R1	NZ_GG688464	439094	Insertion	97.66	128	EFSG_RS08105: Insertion of A at 588	Val197 Frameshift	Capsule EpsG family polymerase (*wzy*)
84R2	NZ_GG688461	441541	SNP	100	157	EFSG_RS08120: 61C ⇒ T	Gln21 ⇒ Stop	Capsule nucleotide sugar dehydrogenase
84R3	NZ_GG688461	441541	SNP	100	138	EFSG_RS08120: 61C ⇒ T	Gln21 ⇒ Stop	Capsule nucleotide sugar dehydrogenase
84R4	NZ_GG688461	441541	SNP	100	128	EFSG_RS08120: 61C ⇒ T	Gln21 ⇒ Stop	Capsule nucleotide sugar dehydrogenase
84R5	NZ_GG688461	436239	Deletion	100	110	EFSG_RS08090: Deletion of C at 641	Ala214 Frameshift	Capsule Aminotransferase

84R6	NZ_GG688461	744354	SNP	100	57	EFSG_RS09545: 557C ⇒ T	Ala186 ⇒ Val	RDD family protein
84R6	NZ_GG688461	431883	SNP	98.92	93	EFSG_RS08070: 250G ⇒ T	Asp84 ⇒ Tyr	Capsule tyrosine-protein kinase (*wze*)
84R8	No mutation detected							

SNP – Single Nucleotide Polymorphism; Indel – Insertion/Deletion; AA – Amino Acid; RDD – Arginine Aspartate Aspartate;

Table S3A. **Phage 9181 resistance enhances antimicrobial susceptibility by E-test**

		Ampicillin		Ceftriaxone		Daptomycin	
**Strain**	**Strain Mutation**	Mean MIC (µg/mL)	Standard deviation (µg/mL)	MIC (µg/mL)	Standard deviation (µg/mL)	MIC (µg/mL)	Standard deviation (µg/mL)
**Com12**	n/a	1.33	0.29	>32	0	1.83	0.29
**81R3**	*sagA*	0.56	0.41	**2.67******	**1.53**	1.83	0.29
**81R4**	*sagA*	**0.5***	**0.25**	**2.67******	**0.58**	1.83	0.29
**81R5**	*sagA*	0.83	0.29	>32	0	1.83	0.29
**81R6**	*sagA*	**0.29****	**0.18**	**0.83******	**0.14**	2.17	0.76
**81R7**	*wze*, *topB*	0.92	0.52	>32	0	1.83	0.29
**81R8**	*sagA*	**0.44***	**0.28**	**1.33******	**0.29**	1.83	0.29

$^{*}P$ < 0.05, $^{**}P$ < 0.01, $^{****}P$ <0.0001 by unpaired t-test; MIC, minimum inhibitory concentration; n/a, not applicable

Table S3B. **Phage 9183 resistance enhances antimicrobial susceptibility by E-test**

		Ampicillin		Ceftriaxone		Daptomycin	
**Strain**	Strain Mutation	MIC (µg/mL)	Standard deviation (µg/mL)	MIC (µg/mL)	Standard deviation (µg/mL)	MIC (µg/mL)	Standard deviation (µg/mL)
**733**	n/a	1.08	0.38	> 32	0	1.67	0.29
**83R1**	*epaR nth*	**0.20***	**0.17**	**0.29******	**0.08**	**0.5****	**0**
**83R3**	*epaR*	**0.36***	**0.16**	**0.33******	**0.14**	**0.42****	**0.14**
**83R4**	*epaX gdh*	**0.34***	**0.19**	**0.33******	**0.14**	**0.33****	**0.14**
**83R5**	*epaR gnsA sorC*	**0.27***	**0.20**	**0.88******	**0.98**	**0.50****	**0.25**
**83R6**	*epaR*	**0.30***	**0.10**	**0.25******	**0**	**0.67***	**0.29**
**83R7**	*epaX*	**0.31***	**0.16**	**0.67******	**0.29**	**0.38****	**0.13**
**83R8**	*epaR*	**0.27***	**0.20**	**0.54******	**0.40**	**0.67***	**0.29**

*$P < 0.05$, **$P < 0.01$, ****$P < 0.0001$ by unpaired t-test; MIC, minimum inhibitory concentration; n/a, not applicable

Table S3C. **Phage 9184 resistance does not alter antimicrobial susceptibility by E-test**

		Ampicillin		Ceftriaxone		Daptomycin	
Strain	Strain Mutation	MIC (µg/mL)	Standard deviation (µg/mL)	MIC (µg/mL)	Standard deviation (µg/mL)	MIC (µg/mL)	Standard deviation (µg/mL)
733	n/a	1.25	0.43	>32	0	1.83	0.29
84R1	*efsg_rs08105*	1.25	0.43	>32	0	1.83	0.29
84R2	*efsg_rs08120*	1.33	0.76	>32	0	1.83	0.29
84R3	*efsg_rs08120*	1.23	0.93	>32	0	2	0
84R4	*efsg_rs08120*	1.17	0.58	>32	0	1.67	0.29
84R5	*efsg_rs08090*	1.17	0.58	>32	0	1.67	0.29
84R6	*rdd*, *wze*	1.33	0.76	>32	0	2	0

MIC, minimum inhibitory concentration; n/a, not applicable

Table S4. Bacterial strains, phages, plasmids and primers

Strains, phages, plasmids and primers	Characteristics and/or description	Reference/ Source
Enterococcus faecium		
**1,141,733**	clinical isolate (blood); USA, 2006	(1)
**Com12**	human fecal isolate; USA, 2006	(1)
**Com15**	Human fecal isolate; USA, 2006	(1)
**1,231,408**	clinical isolate (blood); USA, 2005; Amp^R, Cip^R	(1)
**1,231,501**	clinical isolate (blood); USA, 2005	(1)
**1,231,410**	clinical isolate (blood); USA, 2005; Van^R, Amp^R Cip^R	(1)
**1,231,502**	clinical isolate (blood); USA, 2005; Van^R, Amp^R Cip^R	(1)
**1,230,933**	clinical isolate (blood); USA, 2005; Van^R, Amp^R Cip^R	(1)
**U37**	clinical isolate (tissue source unknown); USA; 1998; Van^R, Amp^R, Erm^R, Gen^R, Str^R, Tet^R	(2)
**H11**	clinical isolate (tissue source unknown); USA; 1998; Van^R, Amp^R, Erm^R, Gen^R, Str^R, Tc^R	(2)
**81R3-*sagA***	81R3 (*sagA* SNP) strain carrying pAM401-*sagA* complementation vector	This study
**81R3-E**	81R3 (*sagA* SNP) strain carrying pAM401 empty vector	This study
**81R4-*sagA***	81R4 (*sagA* SNP) strain carrying pAM401-*sagA* complementation vector	This study
**81R4-E**	81R4 (sagA SNP) strain carrying pAM401 empty vector	This study
**81R5-*sagA***	81R5 (*sagA* SNP) strain carrying pAM401-*sagA* complementation vector	This study
**81R5-E**	81R5 (sagA SNP) strain carrying pAM401 empty vector	This study
**81R6-*sagA***	81R6 (*sagA* SNP) strain carrying pAM401-*sagA* complementation vector	This study
**81R6-E**	81R6 (*sagA* SNP) strain carrying pAM401 empty vector	This study
**81R7-*wze*-Com12**	81R7 (*wze*, *topB* SNPs) strain carrying pLZ12a-*wze*-Com12 complementation vector	This study
**81R7-E**	81R7 (*wze*, *topB* SNPs) strain carrying pLZ12a empty vector	This study
**81R8-*sagA***	81R8 (*sagA* SNP) strain carrying pAM401-*sagA* complementation vector	This study

**81R8-E**	81R8 (*sagA* SNP) strain carrying pAM401 empty vector	This study
**83R1-*epaR***	83R1 (*epaR*, *nth* SNPs) strain carrying pLZ12a-*epaR* complementation vector	This study
**83R1-E**	83R1 (*epaR*, *nth* SNPs) strain carrying pLZ12a empty vector	This study
**83R3-*epaR***	83R1 (*epaR* SNP) strain carrying pLZ12a-*epaR* complementation vector	This study
**83R3-E**	83R3 (*epaR* SNP) strain carrying pLZ12a empty vector	This study
**83R4-*epaX***	83R4 (*epaX, gdh* SNP) strain carrying-*epaX* complementation vector	This study
**83R4-E**	83R4 (*epaX, gdh* SNP) strain carrying pLZ12a empty vector	This study
**83R5-*epaR***	83R5 (*epaR, gnsA*, *sorC* SNPs) strain carrying pLZ12a-*epaR* complementation vector	This study
**83R5-E**	83R5 (*epaR*, *gnsA*, *sorC* SNPs) strain carrying pLZ12a empty vector	This study
**83R6-*epaR***	83R6 (*epaR* SNP) strain carrying pLZ12a-*epaR* complementation vector	This study
**83R6-E**	83R6 (*epaR* SNP) strain carrying pLZ12a empty vector	This study
**83R7-*epaX***	83R7 (*epaX*, *dltA* SNPs) strain carrying pLZ12a-*epaX* complementation vector	This study
**83R7-*dlta***	83R7 (*epaX*, *dltA* SNPs) strain carrying pLZ12a-*dltA* complementation vector	This study
**83R7-E**	83R7 (*epaX*, *dltA* SNPs) strain carrying pLZ12a empty vector	This study
**83R8-*epaR***	83R8 (*epaR* SNP) strain carrying pLZ12a-*epaR* complementation vector	This study
**83R8-E**	83R8 (*epaR* SNP) strain carrying pLZ12a empty vector	This study
**84R2-8120**	84R2 (*efsg_rs08120* SNP) strain carrying pLZ12a-8120 vector	This study
**84R2-E**	84R2 (*efsg_rs08120* SNP) strain carrying pLZ12a empty vector	This study
**84R3-8120**	84R3 (*efsg_rs08120* SP) strain carrying pLZ12a-8120 vector	This study
**84R3-E**	84R3 (*efsg_rs08120* SNP) strain carrying pLZ12a empty vector	This study
**84R4-8120**	84R4 (*efsg_rs08120* SP) strain carrying pLZ12a-8120 vector	This study
**84R4-E**	84R4 (*efsg_rs08120* SNP) strain carrying pLZ12a empty vector	This study
**84R5-8090**	84R5 (*efsg_rs08105* SNP) strain carrying pLZ12a-8090 complementation vector	This study
**84R5-E**	84R5 *(efsg_rs08105* SNP) strain carrying pLZ12a empty vector	This study
**84R6-*wze*-733**	84R6 (*wze*, *rdd* SNP) strain carrying pLZ12a-*wze* complementation vector	This study
**84R6-E**	84R6 (*wze*, *rdd* SNP) strain carrying pLZ12a empty vector	This study

**UCH1**	clinical isolate (blood); Dap^R, Amp^R, Van^R; University of Colorado Hospital	This study
**UCH2**	clinical isolate (blood); University of Colorado Hospital	This study
**UCH3**	clinical isolate (blood); Dap^{SDD}, Amp^R, Van^R; University of Colorado Hospital	This study
**UCH4**	clinical isolate (blood); Dap^R, Amp^R, Van^R; University of Colorado Hospital	This study
**UCH5**	clinical isolate (blood); Dap^R, Amp^R, Van^R; University of Colorado Hospital	This study
**UCH6**	clinical isolate (blood); Dap^R, Lin^I, Amp^R, Van^R; University of Colorado Hospital	This study
**UCH7**	clinical isolate (blood); Dap^{SDD}, Amp^R, Van^R; University of Colorado Hospital	This study
**UCH8**	clinical isolate (blood); Amp^R, Van^R, Str^R; University of Colorado Hospital	This study
**UCH9**	clinical isolate (blood); Amp^R, Van^R; University of Colorado Hospital	This study
**UCH10**	clinical isolate (spleen); Dap^R, Amp^R; University of Colorado Hospital	This study
**UCH11**	clinical isolate (ascites); University of Colorado Hospital	This study
**Phage resistant strains obtained *in vitro***		
**81R3**	phage 9181 resistant mutant of Com12; *sagA* SNP; Trp 433 Leu	This study
**81R4**	phage 9181 resistant mutant of Com12; *sagA* SNP; Trp 433 Cys	This study
**81R5**	phage 9181 resistant mutant of Com12; *sagA* SNP; Gly 460 Asp	This study
**81R6**	phage 9181 resistant mutant of Com12; *sagA* insertion; Phe insertion between Tyr 451 and Leu 452	This study
**81R7**	phage 9181 resistant mutant of Com12; *wze* SNP; Pro 167 Ser; *topB* SNP; Gly 109 Trp	This study
**81R8**	phage 9181 resistant mutant of Com12; *sagA* SNP; Gly 435 Val	This study
**83R1**	phage 9183 resistant mutant of 1,141,733; *epaR* SNP; Arg 338 Cys; *nth* SNP; Ala 10 Ser	This study
**83R2**	Phage 9183 resistant mutat of 1,141,733; unknown mutation causing phage 9183 resistance	This study
**83R3**	phage 9183 resistant mutant of 1,141,733; *epaR* deletion; Phe 103 frameshift	This study
**83R4**	phage 9183 resistant mutant of 1,141,733; *epaX* deletion; Asn 66 frameshift; *gdh* SNP; Trp 104 Cys	This study

**83R5**	phage 9183 resistant mutant of 1,141,733; *epaR* deletion; Glu 211 frameshift; *gnsA* SNP; Glu 2 Gly; *sorC* SNP; Leu 329 Phe	This study
**83R6**	phage 9183 resistant mutant of 1,141,733; *epaR* SNP; Met 310 Ile	This study
**83R7**	phage 9183 resistant mutant of 1,141,733; *epaX* deletion; Asn 66 frameshift; *dltA* SNP; Glu 8 Stop	This study
**83R8**	phage 9183 resistant mutant of 1,141,733; *epaR* SNP; Glu 315 Gly	This study
**84R2**	phage 9184 resistant mutant of 1,141,733; *efsg_rs08120* SNP; Gln 21 Stop	This study
**84R3**	phage 9184 resistant mutant of 1,141,733; *efsg_rs08120* SNP; Gln 21 Stop	This study
**84R4**	phage 9184 resistant mutant of 1,141,733; *efsg_rs08120* SNP; Gln 21 Stop	This study
**84R5**	phage 9184 resistant mutant of 1,141,733; *efsg_rs08090* deletion; Ala 214 frameshift	This study
**84R6**	phage 9184 resistant mutant of 1,141,733; *wze* SNP; Asp 84 Tyr; *rdd* SNP; Ala 186 Val	This study
**84R8**	Phage 9184 resistant mutant of 1,141,733; unknown mutation causing phage 9183 resistance	This study
Enterococcus faecalis		
**OG1RF**	Human oral isolate; Rf^R, Fa^R	(3)
**UCH12**	clinical isolate (blood); Str^R, Gen^R; University of Colorado Hospital	This study
**UCH13**	clinical isolate (blood); Str^R, Gen^R; University of Colorado Hospital	This study
**UCH14**	clinical isolate (blood); Str^R; University of Colorado Hospital	This study
**UCH15**	clinical isolate (blood); University of Colorado Hospital	This study
**UCH16**	clinical isolate (spine tissue); Dox^R; University of Colorado Hospital	This study
**UCH17**	clinical isolate (joint tissue); Dox^R; University of Colorado Hospital	This study
**UCH18**	clinical isolate (heart valve tissue); University of Colorado Hospital	This study
**UCH19**	clinical isolate (heart valve tissue); University of Colorado Hospital	This study
**UCH20**	clinical isolate (heart valve tissue); University of Colorado Hospital	This study
Escherichia coli		

**TG1**	*[F' traD36 proAB lacIqZ ΔM15] supE thi-1 Δ(lac-proAB) Δ(mcrBhsdSM)5(rK - mK -)*	Lucigen
**Phages**		
**phage 9181**	raw sewage isolate, prolate-head, Siphoviridae	This study
**phage 9183**	raw sewage isolate, icosahedral-head, Siphoviridae	This study
**phage 9184**	raw sewage isolate, icosahedral-head, Siphoviridae	This study
**Plasmids**		
**pAM401**	*E. coli*-*E. faecalis* shuttle vector; pIP501 origin; Cm^R, Tc^R	(4)
**pAM401-SagA**	pAM401 plasmid expressing *E. faecium* Com15 *sagA* promoter fused to *sagA* ORF with His-6 tag	(5)
**pLZ12A**	*bacA* promoter cloned into shuttle vector pLZ12; pSH71 origin; Cm^R	(6, 7)
**pLZ12A-*wze*-Com12**	pLZ12A plasmid expressing *E. faecium* Com12 *wze* from the P-*bacA* promoter	This study
**pLZ12A-*epaR***	pLZ12A plasmid expressing *E. faecium* 1,141,733 *epaR* from the *bacA* promoter	This study
**pLZ12A-*epaX***	pLZ12A plasmid expressing *E. faecium* 1,141,733 *epaX* from the *bacA* promoter	This study
**pLZ12A-*dltA***	pLZ12A plasmid expressing *E. faecium* 1,141,733 *dltA* from the *bacA* promoter	This study
**pLZ12A-8120**	pLZ12A plasmid expressing *E. faecium* 1,141,733 *efsg_rs08120* from the *bacA* promoter	This study
**pLZ12A-8090**	pLZ12A plasmid expressing *E. faecium* 1,141,733 *efsg_rs08090* from the *bacA* promoter	This study
**pLZ12A-*wze*-733**	pLZ12A plasmid expressing *E. faecium* 1,141,733 *wze* from the *bacA* promoter	This study
**Primers**		
***wze*-Com12-comp-F**	NNNNNNGAATTCATGGCACGAACACAGAAACA	This Study

***wze*-Com12-comp-R**	NNNNNNGGATCCTCGGTGGATGTCTTCGATCA	This Study
***epaR*-comp-F**	NNNNNNCTGCAGATGAATAAAAATGGGGAGTGGAATG	This Study
***epaR*-comp-R**	NNNNNNGGATCCCTCCTTGGATAGCTGACTGAATC	This Study
***epaX*-comp-F**	NNNNNNGAATTCATGTGTGAGATTAGTATTATTGTTCCTG	This Study
***epaX*-comp-R**	NNNNNNGGATCCTGAAATGGTCCTCCCTACCT	This Study
***dltA*-comp-F**	NNNNNNGAATTCATGGAAATCAAAACGATTATTGAAGC	This Study
***dlta*-comp-R**	NNNNNGGATCCAACGATTGGTATAAGCGCAATG	This Study
**8120-comp-F**	NNNNNNCTGCAGATGAAAGTATCAGTTTTTGGTCTC	This Study
**8120-comp-R**	NNNNNNGGATCCTTCATGGTTTAATCCCGTCTAA	This Study
**8090-comp-F**	NNNNNNGAATTCTTGGAAAATAAACGAATATTATTAGCATCT	This Study
**8090-comp-R**	NNNNNNGGATCCTGAGAGCGATACTTGACAATAGG	This Study
***wze*-733-comp-F**	NNNNNNGAATTCATGGCACGAACACAGAAACA	This Study
***wze*-733-comp-R**	NNNNNNGGATCCCGATCATTTCTCTTGTCTCCTCTC	This Study
**16270-Com12-gap-F**	NNNNNNCAGAACAAGCACGTCAACAAG	This Study
**16270-com12-gap-R**	NNNNNNAATCCTGAGCAGTCAAATCCA	This Study
**Phage-9181-Lysin-F**	GCAACGCATAACCAACCTAAC	This Study
**Phage-9181-Lysin-R**	GTCTCCACCTTGATAGCCATAC	This Study
**Phage-9183-Integrase-F**	GCAGACATTCGTGCTTTCTTT	This Study
**Phage-9183-Integrase-R**	CTCCTCGTTGATCAAACCATTTC	This Study
**Phage-9184-Lysin-F**	GGGTAACTCAACAGCCATACA	This Study
**Phage-9184-Lysin-R**	AGTTCTTGTCCGCCTTGATAG	This Study

Dap^R - daptomycin resistance; Dap^{SDD} - daptomycin susceptibility dose dependent; Lin^I - linezolid intermediate; Amp^R - ampicillin resistant; Van^R - vancomycin resistant; Str^R - Streptomycin resistant; Erm^R – erythromycin resistant; Gen^R – gentamicin resistant; Cip^R – ciprofloxacin resistant; Dox^R – doxycycline resistant; Rf^R – rifampin resistant; Fa^R – fusidic acid resistant; Restriction sites are underlined

References:

1. Palmer KL, Godfrey P, Griggs A, Kos VN, Zucker J, Desjardins C, Cerqueira G, Gevers D, Walker S, Wortman J, Feldgarden M, Haas B, Birren B, Gilmore MS. 2012. Comparative genomics of enterococci: variation in *Enterococcus faecalis*, clade structure in *E. faecium*, and defining characteristics of *E. gallinarum* and *E. casseliflavus*. mBio 3:e00318-11. doi:10.1128/mBio.00318-11.

2. Rice LB, Carias LL, Donskey CL, Rudin SD. 1998. Transferable, plasmid-mediated VanB-type glycopeptide resistance in *Enterococcus faecium*. Antimicrob Agents Chemother 42:963-4.

3. Bourgogne A, Garsin DA, Qin X, Singh KV, Sillanpaa J, Yerrapragada S, Ding Y, Dugan-Rocha S, Buhay C, Shen H, Chen G, Williams G, Muzny D, Maadani A, Fox KA, Gioia J, Chen L, Shang Y, Arias CA, Nallapareddy SR, Zhao M, Prakash VP, Chowdhury S, Jiang H, Gibbs RA, Murray BE, Highlander SK, Weinstock GM. 2008. Large scale variation in *Enterococcus faecalis* illustrated by the genome analysis of strain OG1RF. Genome Biol 9:R110. doi:10.1186/gb-2008-9-7-r110.

4. Wirth R, An FY, Clewell DB. 1986. Highly efficient protoplast transformation system for *Streptococcus faecalis* and a new *Escherichia coli*-*S. faecalis* shuttle vector. J Bacteriol 165:831-6. doi:10.1128/jb.165.3.831-836.1986.

5. Rangan KJ, Pedicord VA, Wang YC, Kim B, Lu Y, Shaham S, Mucida D, Hang HC. 2016. A secreted bacterial peptidoglycan hydrolase enhances tolerance to enteric pathogens. Science 353:1434-1437. doi:10.1126/science.aaf3552.

6. Chatterjee A, Johnson CN, Luong P, Hullahalli K, McBride SW, Schubert AM, Palmer KL, Carlson PE, Jr., Duerkop BA. 2019. Bacteriophage resistance alters antibiotic-mediated intestinal expansion of enterococci. Infect Immun 87:e00085-19. doi:10.1128/iai.00085-19.

7. Perez-Casal J, Caparon MG, Scott JR. 1991. Mry, a trans-acting positive regulator of the M protein gene of *Streptococcus pyogenes* with similarity to the receptor proteins of two-component regulatory systems. J Bacteriol 173:2617-24. doi:10.1128/jb.173.8.2617-2624.1991.
